# Supplementary material for: Novel synthesis of topological insulator based nanostructures (Bi2Te3) demonstrating high performance photodetection
Source: Sci Rep. 2019 Mar 7;9:3804. doi: 10.1038/s41598-019-40394-z (PMC6405830; doi:10.1038/s41598-019-40394-z)
Supplement: Supplementary file 1 — Supplementary Info [file 41598_2019_40394_MOESM1_ESM.pdf]

Supplementary Information

Novel synthesis of topological insulator based nanostructures (Bi_2Te_3) demonstrating high performance photodetection

Alka Sharma[1,2], T D Senguttuvan[1,2], V N Ojha[1,2] and Sudhir Husale[1,2]*

[1]Academy of Scientific and Innovative Research (AcSIR), National Physical Laboratory, Council of Scientific and Industrial Research, Dr. K. S Krishnan Road, New Delhi-110012, India.

[2] National Physical Laboratory, Council of Scientific and Industrial Research, Dr. K. S Krishnan Road, New Delhi-110012, India.

*E-mail: husalesc@nplindia.org

Supplementary Information includes:

1. Fig S1, Bi_2Te_3 nanostructure growth on glass, quartz and saphhire

2. Fig S2. Growth of Bi_2Te_3 quintuples, dots and nanocrystals

3. Fig S3 HRTEM characterization of Bi_2Te_3 nanosheets

4. Fig S4 Raman characterization of Bi_2Te_3 nanosheets

5. Fig S5 Deposition efficiency on SiO_2, Au, SI_3N_4, ITO, ebeam patterned and FIB milled substrates

6. Fig S6 Broadspectral photoresponse of different nanostructure devices.

7. Fig S7. Rise and decay time curve fitting

8. Table I. Synthesis comparison of topological insulator nanostructures using various techniques

9. Table II. Photorepsonse properties of topological insulator based nanostructures and thin films.

1. Fig S1. Bi_2Te_3 nanostructure growth on glass, quartz and saphhire

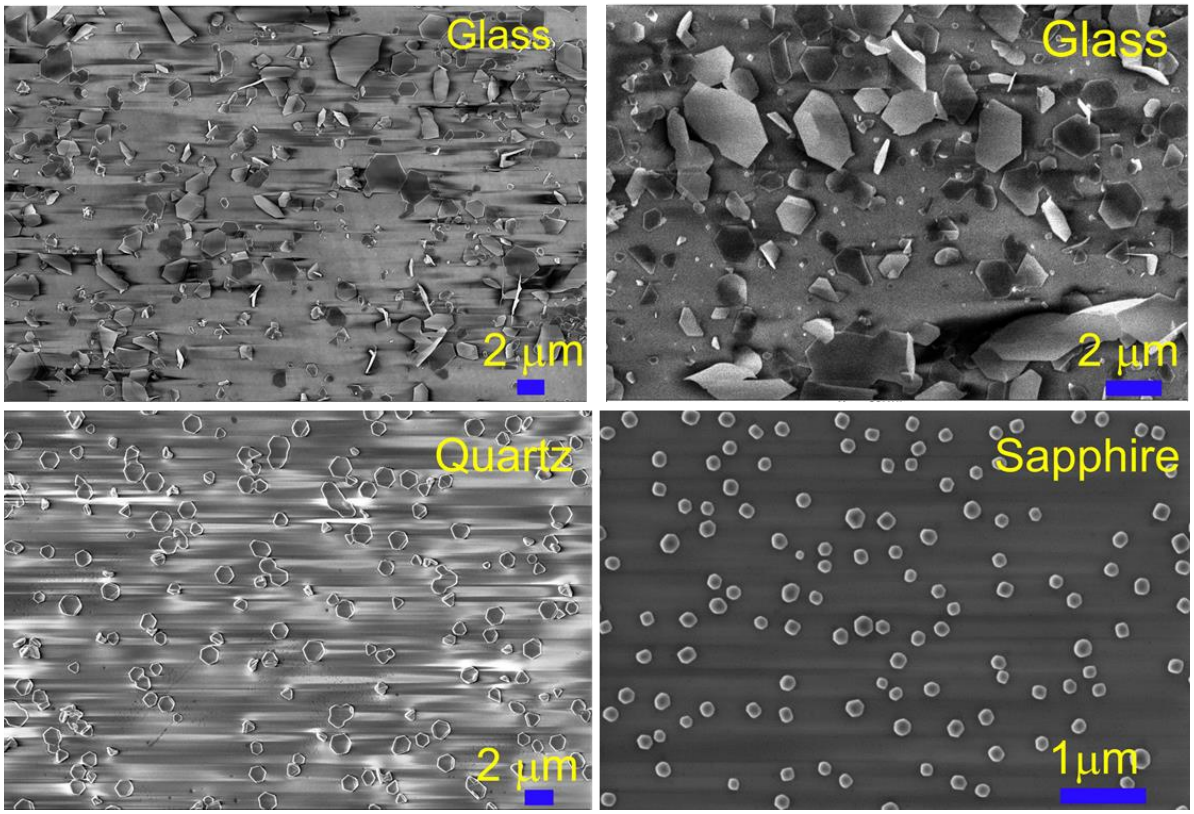

2. Fig S2. Growth of Bi_2Te_3 quintuples, dots and nanocrystals

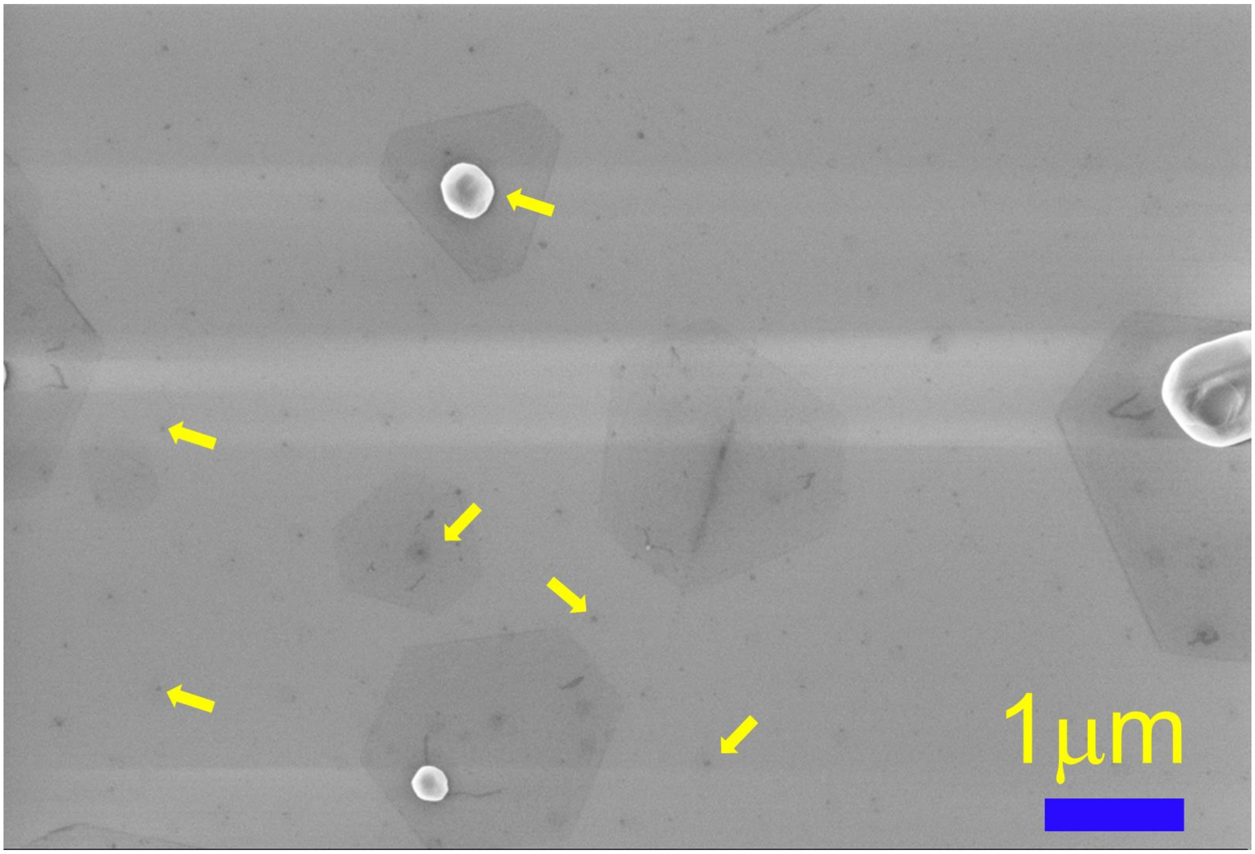

3. Fig S3. HRTEM characterization of Bi_2Te_3 nanosheets

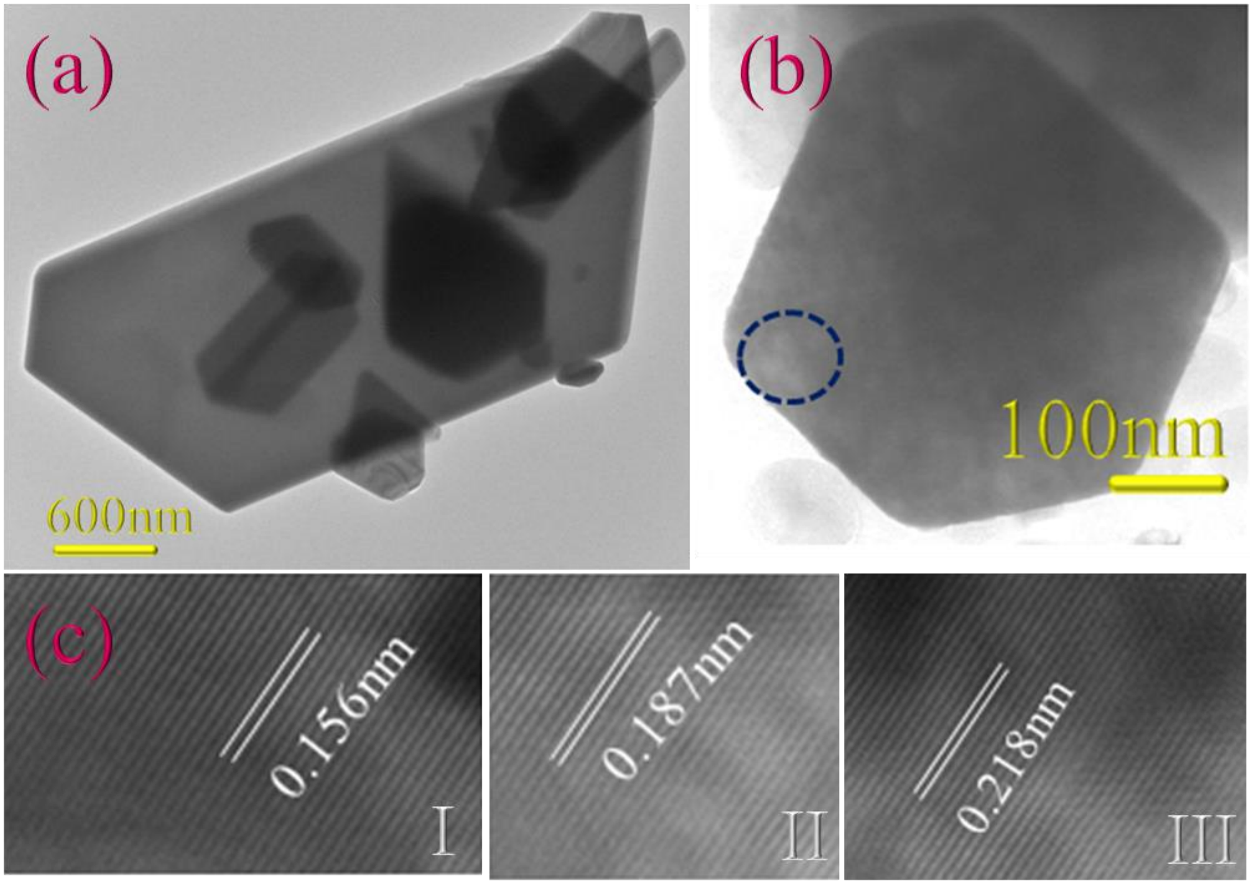

Fig.S3 HRTEM images of Bi_2Te_3 nanosheets. (a,b) Bright field high resolution micrograph of the Bi_2Te_3 nanostructures showing different shapes- hexagon, triangle, pentagon etc. (c I-III), atomic scale images of the location shown in Fig.S2 (b) by dotted circle.

The high resolution transmission electron microscopy (HRTEM, model Tecnai G2F30 STWIN) was used to determine the crystalline nature of Bi_2Te_3 nanostructures. HRTEM images shown in S3 (a) indicate that the nanostructures possess sharp edges and elongated or flat morphologies. The HRTEM micrograph of Bi_2Te_3 nanostructure clearly shows the atomic planes of Bi_2Te_3 with interplaner distance (d) 0.156, 0.187 and 0.218 nm.

4. Fig S4. Raman characterization of Bi2Te3 nanosheet

Following fig shows the Raman spectrum of the Bi_2Te_3 nanosheet grown on Ti substrate. The three characteristics vibration modes peaks of Bi_2Te_3 nanosheet are labelled with A_g^1 , E_g^2 and A_g^2.

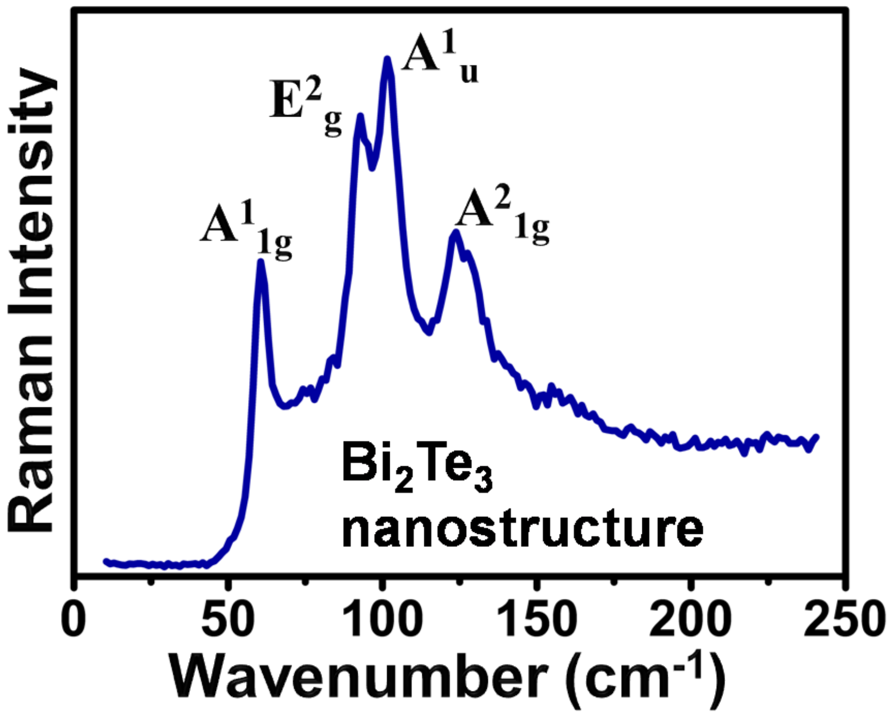

Fig.S4 Raman spectrum of hexagon shaped Bi_2Te_3 nanostructure grown on Ti substrate with vibration modes ${A^1}_{1g}$, E_g^2, A_u^1 and ${A^2}_{1g}$.

5. Fig S5 Deposition efficiency on SiO_2, Au, SI_3N_4, ITO, ebeam patterned and FIB milled substrates

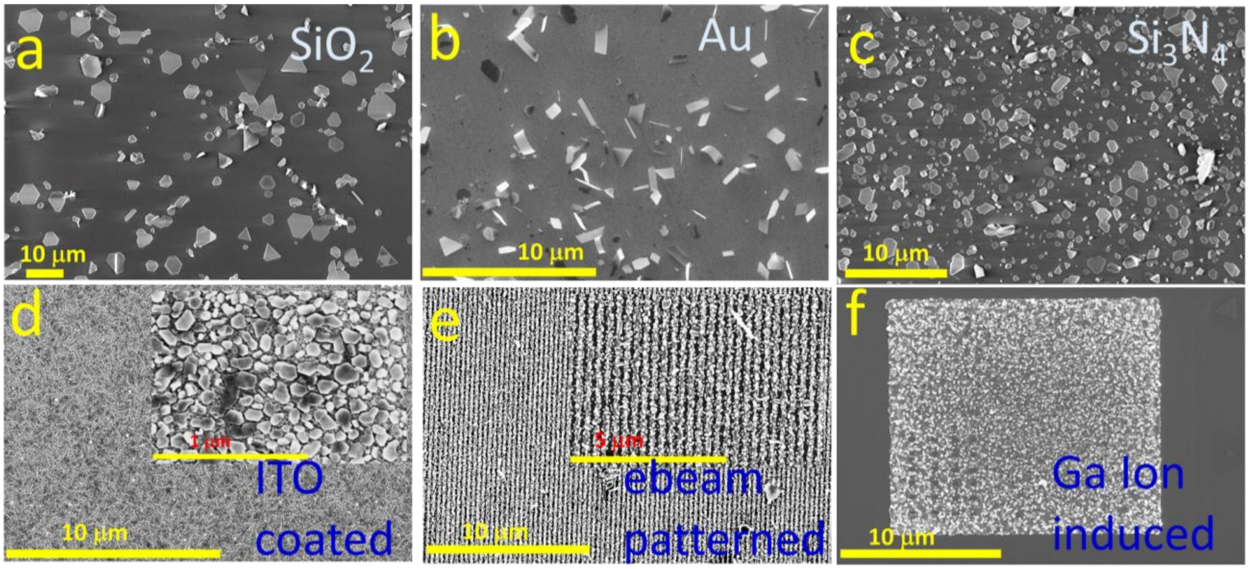

Supplementary information Figure S5 (a-f) represents the different deposition efficiencies on various substrates.

6. Fig S6 Broadspectral photoresponse of different nanostructure devices.

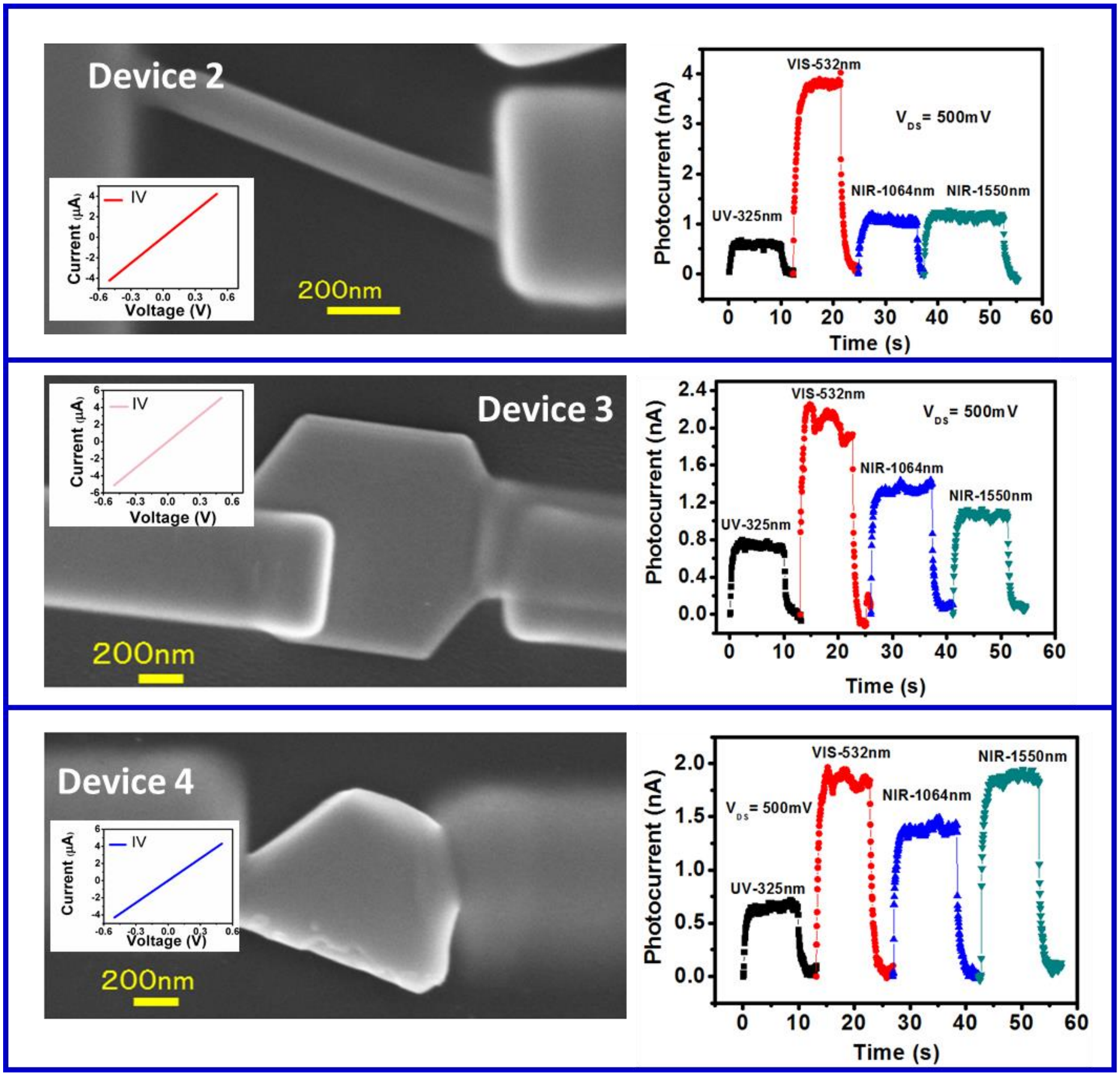

7. Fig S7. Rise and decay time curve fitting

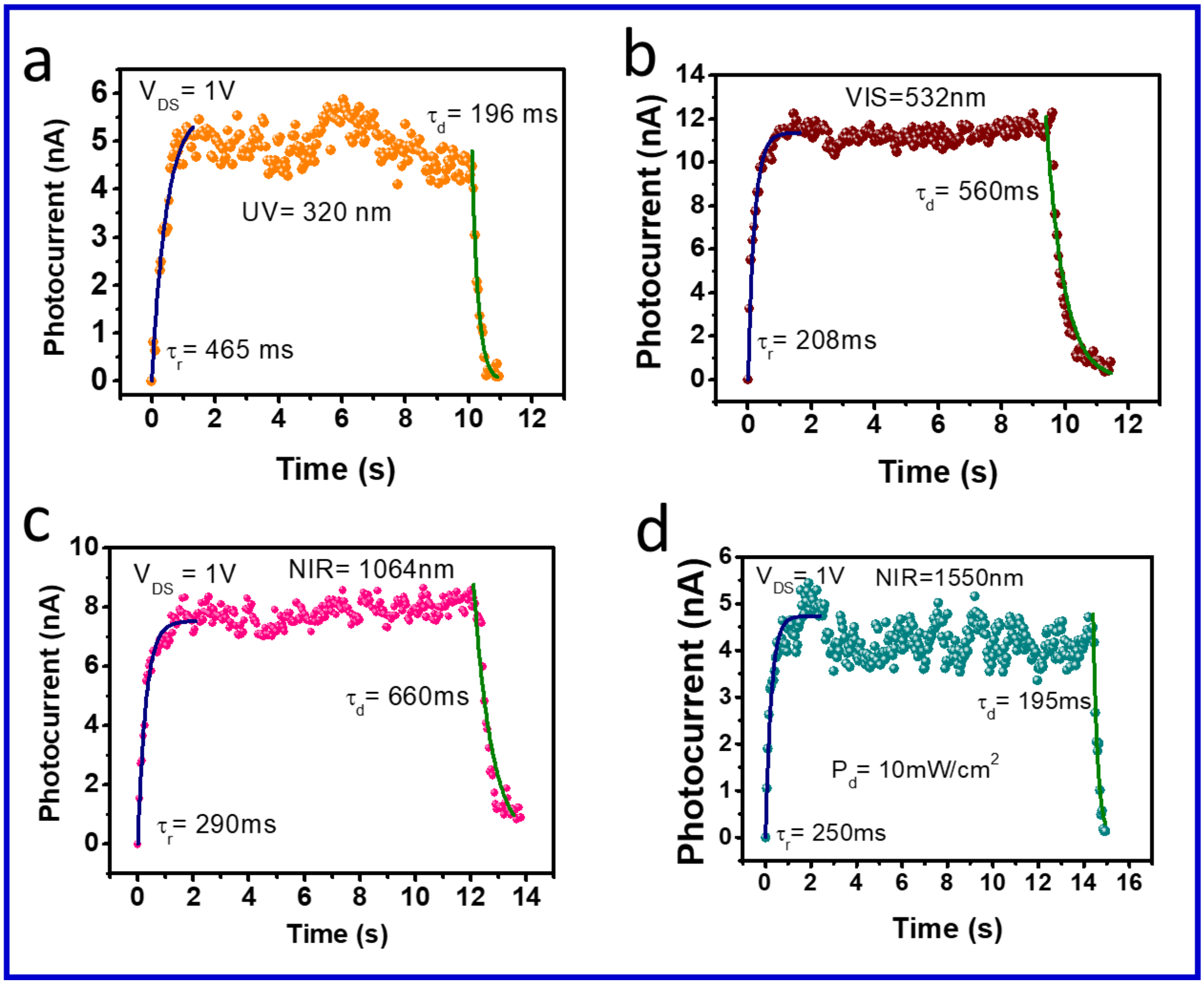

8. Table I. Synthesis comparison of topological insulator nanostructures using various techniques

**Synthesis Method**	**Advantages**	**Disadvantages**	**Ref.**
Exfoliation by mechanical (scotch tape)/chemical (lithium) technique	Simple, cost effective, clean and pristine samples	Irregular shapes and sizes of the flakes, low deposition yield, no control on the thickness of the exfoliated flakes, poor reproducibility	[1,2]
VLS (Vapor liquid solid)	High quality of nanomaterials and mostly moderate deposition efficiency, synthesis of complex nanostructures	Mostly slow growth, need catalytic particles, unidirectionality, cost-effectiveness, no control over crystal morphologies	[3,4]
VS (Vapor solid growth)	Catalyst free, high quality of nanomaterials,<br>Device friendly substrate, synthesis of complex nanostructures	Deposition efficiency, growth control on specific shapes, cost-effectiveness	[5,6]
MOCVD (metal organic chemical vapor deposition)	Concn control of Bi and Te during depostion	Expensive method, chemical based, low deposition efficiency yield	[7,8]
MBE (molecular beam epitaxy)	Highest purity in grown samples, film thickness control, easy doping	Highly expensive, high maintenance costs, high vacuum is needed, time consuming, limited for selective substrates and different types of nanostructures.	[9,10]
Solvothermal Synthesis	Diverse morphological structures can be synthesized	Chemical and precursor based, not device friendly, uneven solution temp, yield and low purity	[11,12]
Pulsed laser deposition (PLD)	Useful as versatile approach, different material combinations	Not useful for large area deposition, uneven coverage, particulate concentration	[13,14]
Confined thin film melting deposition :<br>Compared to above techniques our method include i) single step approach (assuming source and deposition substrate available), ii) growth of nanostructures on device friendly substrates, iii) do not require lattice matching, iv) demonstration of scalable synthesis (on texture substrate ITO), v) simplest way to form hetrostructures , vi) precursor and catalyst free and vii) cost effectiveness.<br>**Future perspective:** The cassette structure based confined melting used in this method just depends on the melting temperature of the material used. There are many novel TMDs (e.g. metal Mo, W and chalcogen atoms S, Se, Te), TI materials beyond the wonder material graphene, those have temperature less than 1200 °C and hence nanostructures of these materials can be synthesized by using this technique in the future where one needs just thin film of such material and hence this method can be used as substitute if compared with the techniques like VLS, CVD or MBE.			Our Work

9. Table II. Photorepsonse properties of topological insulator based nanostructures and thin films.

**Material**	**λ (nm)**	**R (AW^{-1})**	**D (Jones)**	**Gain/EQE**	**Rise/Decay τ_r / τ_d, (s)**
SnTe[15]	405-3800	3.75	-------	-----------	0.31/085
Bi_2Se_3 nanosheets[16]	-------	20.48 x 10^{-3}	---------	8.36	0.7/1.48
SnTe/Si[17]	300-1100	2.36	1.54 x 10^{14}	---	2.2 x10^{-6}/3.8 x10^{-6}
Sb_2Te_3 film[18]	980	21.7	1.22 x 10^{11}	27.4	238.7 / 203.5
Bi_2Te_3 NW[19]	532	251 ±0.32	4.5 x 10^9	586.15±0.1	0.48/0.54
	325	26.82±0.33	1.29 x 10^9	102±0.46	0.28 / 1.6
Bi film[20]	370	250 x 10^{-3}	----------	----------	0.9/1.9
Bi_2Se_3 film / Si[21]	808	924.2	2.38 x 10^{12}	1421	0.045/0.047
Bi_2Te_3-Graphene[22]	532	35	----------	83	8.7x10^{-3}/ 14.8x10^{-3}
	980	10		11	
Bi_2Se_3 nanowire (NW) [23]	1064	300	7.5 x 10^9	350	0.550/0.400
Bi_2Se_3 (NW)/ Si[24]	808	24.28	4.39 x10^{12}	37.4	2.5 x 10^{-6} / 5.5 x 10^{-6}
Polycrystalline Bi_2Te_3 / Si [25]	635	1	2.5 x 10^{11}	-----------	0.1/0.1
WS_2 -Bi_2Te_3[26]	370-1550	30.4	2.3 x 10^{11}	---------	0.020 / 0.020

References:

1 Cho, S. J., Butch, N. P., Paglione, J. & Fuhrer, M. S. Insulating Behavior in Ultrathin Bismuth Selenide Field Effect Transistors. *Nano Lett.* **11**, 1925-1927, doi:10.1021/nl200017f (2011).

2 Ren, L. *et al.* Large-scale production of ultrathin topological insulator bismuth telluride nanosheets by a hydrothermal intercalation and exfoliation route. *J. Mater. Chem.* **22**, 4921-4926, doi:10.1039/c2jm15973b (2012).

3 Meister, S. *et al.* Synthesis and characterization of phase-change nanowires. *Nano Lett.* **6**, 1514-1517, doi:10.1021/nl061102b (2006).

4 Wang, Z. H., Li, M. Z., Yang, L., Zhang, Z. D. & Gao, X. P. A. Broadband photovoltaic effect of n-type topological insulator Bi2Te3 films on p-type Si substrates. *Nano Res.* **10**, 1872-1879, doi:10.1007/s12274-016-1369-2 (2017).

5 Fang, L. *et al.* Catalyst-Free Growth of Millimeter-Long Topological Insulator Bi2Se3 Nanoribbons and the Observation of the pi-Berry Phase. *Nano Lett.* **12**, 6164-6169, doi:10.1021/nl302989v (2012).

6 Tu, N. H. *et al.* Large-Area and Transferred High-Quality Three-Dimensional Topological Insulator Bi2-xSbxTe3-ySey Ultrathin Film by Catalyst-Free Physical Vapor Deposition. *Nano Lett.* **17**, 2354-2360, doi:10.1021/acs.nanolett.6b05260 (2017).

7 Alegria, L. D. & Petta, J. R. Controlled MOCVD growth of Bi2Se3 topological insulator nanoribbons. *Nanotechnology* **23**, doi:10.1088/0957-4484/23/43/435601 (2012).

8 Cao, H. L. *et al.* Topological insulator Bi2Te3 films synthesized by metal organic chemical vapor deposition. *Appl.Phy. Lett.* **101**, doi:10.1063/1.4760226 (2012).

9 Chen, X., Ma, X. C., He, K., Jia, J. F. & Xue, Q. K. Molecular Beam Epitaxial Growth of Topological Insulators. *Adv. Mater.* **23**, 1162-1165, doi:10.1002/adma.201003855 (2011).

10 Li, Y. Y. *et al.* Intrinsic Topological Insulator Bi2Te3 Thin Films on Si and Their Thickness Limit. *Adv. Mater.* **22**, 4002-4007, doi:10.1002/adma.201000368 (2010).

11 Wang, W. Z., Poudel, B., Yang, J., Wang, D. Z. & Ren, Z. F. High-yield synthesis of single-crystalline antimony telluride hexagonal nanoplates using a solvothermal approach. *J. Am. Chem. Soc.* **127**, 13792-13793, doi:10.1021/ja054861p (2005).

12 Zhu, H. T. *et al.* Tri-wing bismuth telluride nanoribbons with quasi-periodic rough surfaces. *J. Mater. Chem.* **21**, 12375-12380, doi:10.1039/c1jm11715g (2011).

13 Yao, J. D. & Yang, G. W. Flexible and High-Performance All-2D Photodetector for Wearable Devices. *Small* **14**, doi:10.1002/smll.201704524 (2018).

14 Yao, J. D., Shao, J. M., Li, S. W., Bao, D. H. & Yang, G. W. Polarization dependent photocurrent in the Bi2Te3 topological insulator film for multifunctional photodetection. *Sci. Rep.* **5**, doi:10.1038/srep14184 (2015).

15 Jiang, T. *et al.* Broadband High-Responsivity Photodetectors Based on Large-Scale Topological Crystalline Insulator SnTe Ultrathin Film Grown by Molecular Beam Epitaxy. *Adv. Opt. Mater.* **5**, doi:10.1002/adom.201600727 (2017).

16 Zang, C. *et al.* Photoresponse properties of ultrathin Bi2Se3 nanosheets synthesized by hydrothermal intercalation and exfoliation route. *Appl. Surf. Sci.* **316**, 341-347, doi:10.1016/j.apsusc.2014.07.064 (2014).

17 Zhang, H. B., Man, B. Y. & Zhang, Q. Topological Crystalline Insulator SnTe/Si Vertical Heterostructure Photodetectors for High-Performance Near-Infrared Detection. *ACS App. Mater. & Int.* **9**, 14067-14077, doi:10.1021/acsami.7b01098 (2017).

18 Zheng, K. *et al.* Optoelectronic characteristics of a near infrared light photodetector based on a topological insulator Sb2Te3 film. *J. Mater.Chem. C* **3**, 9154-9160, doi:10.1039/c5tc01772f (2015).
19 Sharma, A., Srivastava, A., Senguttuvan, T. & Husale, S. Robust broad spectral photodetection (UV-NIR) and ultra high responsivity investigated in nanosheets and nanowires of Bi 2 Te 3 under harsh nano-milling conditions. *Sci. Rep.* **7**, 17911 (2017).
20 Yao, J. D., Shao, J. M. & Yang, G. W. Ultra-broadband and high-responsive photodetectors based on bismuth film at room temperature. *Sci. Rep.* **5**, doi:10.1038/srep12320 (2015).
21 Liu, C. *et al.* Topological insulator Bi2Se3 nanowire/Si heterostructure photodetectors with ultrahigh responsivity and broadband response. *J. Mater. Chem. C* **4**, 5648-5655, doi:10.1039/c6tc01083k (2016).
22 Qiao, H. *et al.* Broadband Photodetectors Based on Graphene-Bi2Te3 Heterostructure. *ACS Nano* **9**, 1886-1894, doi:10.1021/nn506920z (2015).
23 Sharma, A., Bhattacharyya, B., Srivastava, A. K., Senguttuvan, T. D. & Husale, S. High performance broadband photodetector using fabricated nanowires of bismuth selenide. *Sci. Rep.* **6**, doi:10.1038/srep19138 (2016).
24 Zhang, H. B., Zhang, X. J., Liu, C., Lee, S. T. & Jie, J. S. High-Responsivity, High-Detectivity, Ultrafast Topological Insulator Bi2Se3/Silicon Heterostructure Broadband Photodetectors. *ACS Nano* **10**, 5113-5122, doi:10.1021/acsnano.6b00272 (2016).
25 Yao, J. D., Shao, J. M., Wang, Y. X., Zhao, Z. R. & Yang, G. W. Ultra-broadband and high response of the Bi2Te3-Si heterojunction and its application as a photodetector at room temperature in harsh working environments. *Nanoscale* **7**, 12535-12541, doi:10.1039/c5nr02953h (2015).
26 Yao, J. D., Zheng, Z. Q. & Yang, G. W. Layered-material WS2/topological insulator Bi2Te3 heterostructure photodetector with ultrahigh responsivity in the range from 370 to 1550 nm. *J. Mater. Chem. C* **4**, 7831-7840, doi:10.1039/c6tc01453d (2016).
